# Multivariate analysis of prognostic factors in patients with nodular melanoma

**DOI:** 10.1007/s00432-021-03562-1

**Published:** 2021-02-25

**Authors:** L. Susok, M. Stücker, F. G. Bechara, E. Stockfleth, T. Gambichler

**Affiliations:** Department of Dermatology, Ruhr-University Bochum, Gudrunstraße 56, 44791 Bochum, Germany

**Keywords:** Nodular melanoma, Superficial spreading melanoma, Tumor thickness, Prognostic, Factors, Logistic regression

## Abstract

**Purpose:**

Nodular melanoma (NM) is associated with worse disease outcome when compared to superficial spreading melanoma (SSM). We aimed to perform a single-center analysis of prognostic factors in patients with NM and compare the data with SSM patients.

**Methods:**

We studied 228 patients with NN and 396 patients with SSM. Patients with in situ melanomas or stage IV at diagnosis were not included in the study. Data were analyzed using the Mann–Whitney test, Chi-square test, Kaplan–Meier curves including the log-rank test, and logistic regression model.

**Results:**

When compared to patients with SSM, patients with NM had less likely lower Clark level, higher tumor thickness, less likely tumor regression, more often ulcerated tumors, and less likely a history of precursor lesions such as a nevus. Within a 5-year follow-up we observed significantly more disease relapses and deaths in NM patients than in SSM patients. On multivariate analysis, disease relapse in NM patients was independently predicted by tumor thickness and positive SLNB, whereas melanoma-specific death of NM patients was independently predicted by male sex and tumor thickness. Histologic regression also remained in the logistic regression model as a significant independent negative predictor of NM death.

**Conclusions:**

We did not observe that NM subtype was per se a significant independent predictor for disease relapse or melanoma-specific death. Among the well-known prognostic factors such as tumor thickness and male sex, NM is also associated with other unfavorable factors such as absence of regression.

## Introduction

In Caucasians, incidences of malignant melanoma (MM) are increasing worldwide, with estimated continuous case increases for the next decades. The highest incidence is found in Queensland, Australia (about 70 cases/100.000/year). In the USA, an increasing incidence from 14 to 22/100.000 person-years has been observed across all primary tumor thicknesses. Similarly, the incidence of invasive MM increases in Europe mostly attributed to the increasing incidence of thin melanomas.^1–3^ MM is a heterogeneous neoplasm that is usually classified into four major subtypes: superficial spreading melanoma (SSM), nodular melanoma (NM), lentigo maligna melanoma, and acral lentiginous melanoma, whereby the two most common subtypes are SSM (about 65% of cases) and NM (about 15% of cases). Histologically, a mainly epidermal portion with slow horizontal growth pattern is characteristic for SSM. In contrast, NM is mostly thicker than SSM due to the lack of a significant intra-epidermal portion and is characterized by a quick vertical growth pattern. SSM and NM are representatives of MM progression, which is perceived as a stepwise process starting with healthy melanocytes at the epidermal–dermal junction getting mutations that results to radial growth-phase MM to vertical growth-phase MM and, finally, metastatic disease. Nevertheless, clinicopathologic and epidemiologic data of many research groups give support to the view that SSM and NM progress independently. In contrast to the general notion that NM is associated with worse disease outcome only due to its higher tumor thickness of the primary, recent data indicate that the risk of NM may be based on more aggressive biological features (Whiteman et al. 2016; Dessinioti et al. 2019, 2018; Lattanzi et al. 2019; Mar et al. 2013; Sacchetto et al. 2018; Shaikh et al. 2012). We aimed to perform a large single-center analysis of prognostic factors in patients with NM and compare the data with SSM patients.

## Materials and methods

### Patients

We searched our institutional melanoma database for NN and SSM patients. All patients had been treated at the Skin Cancer Center of the Department of Dermatology (Ruhr-University Bochum, Germany) between July 2001 and August 2011. The study population included 228 patients with NN and 396 patients with SSM (Table 1). Patients with in situ melanomas or stage IV at first diagnosis were not included in the analysis. Patient data, including gender, age, tumor evolution, tumor thickness and high-risk tumor thickness (≥ 2 mm), ulceration, regression, etc., were collected from the electronic records. Patients were staged or re-staged according to the final version of the 2009 AJCC melanoma staging and classification system (Balch et al. 2009). All primary tumors were examined by at least two senior dermato-histopathologists of the Skin Cancer Center of the Department of Dermatology (Ruhr-University Bochum). Immunohistochemistry was carried out with antibodies against S100B and Melan-A/MART-1, and in ambiguous cases also with HMB45 and Ki-67 (DAKO, Hamburg, Germany).

**Table 1:** Comparison of nodular melanoma (NM) patients and superficial spreading melanoma (SSM) patients (univariate analyses)

Parameters	NM *n* = 228	SSM *n* = 396	*P* value Mann–Whitney, Chi, log-rank test
Age median (range) years	69.5 (27–97)	68 (20–94)	0.32
Gender f/m	113/115 (49.6%/50.4%)	213/183 (53%/46.2%)	0.79
Location			
Head/neck	5 (2.2%)	17 (4.3%)	0.78
Upper limbs	48 (21.1%)	61 (15.4%)	
Lower limbs	67 (29.4%	126 (31.8%)	
Trunk	108 (47.4%)	192 (48.5%)	
Clark level			
II	2 (0.9%)	22 (5.6%)	< 0.0001
III	50 (21.9%)	112 (28.3%)	
IV	161 (70.6%)	258 (65.2%)	
V	15 (6.6%)	4 (1%)	
Median tumor thickness mm	1.6 (0.2–15)	1.4 (0.4–7)	
High-risk melanoma (> 2 mm thickness)			
No/yes	88/140 (38.6%/61.4%)	320/76 (80.8%/19.2%)	< 0.0001
Regression			
No/yes	218/10 (95.6%/4.4%)	345/51 (87.1%/12.9%)	0.019
Ulceration			
No/yes	115/113 (50.4%/49.6%)	299/97 (75.5%/24.5%)	0.0008
Evolution of melanoma			
No precursor lesion/Precursor lesion	115/113 (50.4%/49.6%)	45/351 (11.4%/88.6%)	< 0.0001
Positive sentinel lymph node biopsy			
No/yes	140/88 (61.4%/38.6%)	327/69 (82.2%/17.4%)	< 0.0001
Melanoma stage*			
IA	4 (1.8%)	44 (11.1%)	< 0.0001
IB	42 (18.4%)	196 (49.5%)	
IIA	39 (17.1%)	65 (16.4%)	
IIB	40 (17.5%)	18 (4.5%)	
IIC	19 (8.3%)	4 (1%)	
IIIA	44 (19.3%)	36 (9.1%)	
IIIB	28 (12.3%)	22 (5.6%)	
IIIC	12 (5.2%)	11 (2.8%)	
Adjuvant interferon			
No/yes	115/113 (50.4%/49.6%)	281/115 (71%/29%)	< 0.0001
5-year disease relapse			
No/yes	141/87 (61.8%/39.2%)	299/97 (75.5%/24.5%)	< 0.0001
5-year melanoma-specific death			
No/yes	159/69 (69.7%/30%)	324/72 (81.8%/18.2%)	0.0004

*AJCC 2009

### Treatment

The management of patients was performed guideline-adjusted according to the tumor stage (Garbe et al. 2006). All tumors were treated by primary excision including safety margin. Predominant indication for sentinel lymph node biopsy (SLNB) was a Breslow tumor thickness of 1 mm or more. Upgrading of tumors less than 1 mm was considered in the presence of a Clark level of IV or higher and ulceration. Prior to SLNB, evidence of macro-metastatic disease in regional lymph nodes or distant sites was ruled out by physical examination, imaging with computed tomography, etc. Patients with metastatic regional lymph nodes were subjected to complete lymph node dissection. All patients with a primary melanoma thickness of 1.5 mm or more were considered for adjuvant low-dose interferon alfa-2b (Roferon; Roche Pharma AG, Grenzach-Wyhlen, Germany) therapy, and patients with melanoma-positive lymph nodes were considered for adjuvant high-dose interferon (Intron; MSD, Munich, Germany) therapy. Metastatic disease was usually treated with mono-dacarbacine, mono-temozolomide, or polychemotherapy using gemcitabine/treosulfan or carboplatin/paclitaxel (Garbe et al. 2006). Follow-up data were collected using chart review and contacting patients, relatives, and resident practitioners and dermatologists. The study was approved by the Ethics Committee of the Ruhr-University Bochum (#4749-13) and conducted according to the principles of the Declaration of Helsinki.

### Statistics

Data analysis was performed using the statistical package MedCalc Software version 19.6.1 (MedCalc, Ostend, Belgium). Distribution of data was assessed by the D`Agostino-Pearson test. For non-normally distributed data, the median and range were calculated. Data were analyzed using the Mann–Whitney test, Chi-square test, Kaplan–Meier curves including the log-rank test, and logistic regression model using stepwise data inclusion. *P* values smaller than 0.05 were considered significant.

## Results

**Fig. 1:**
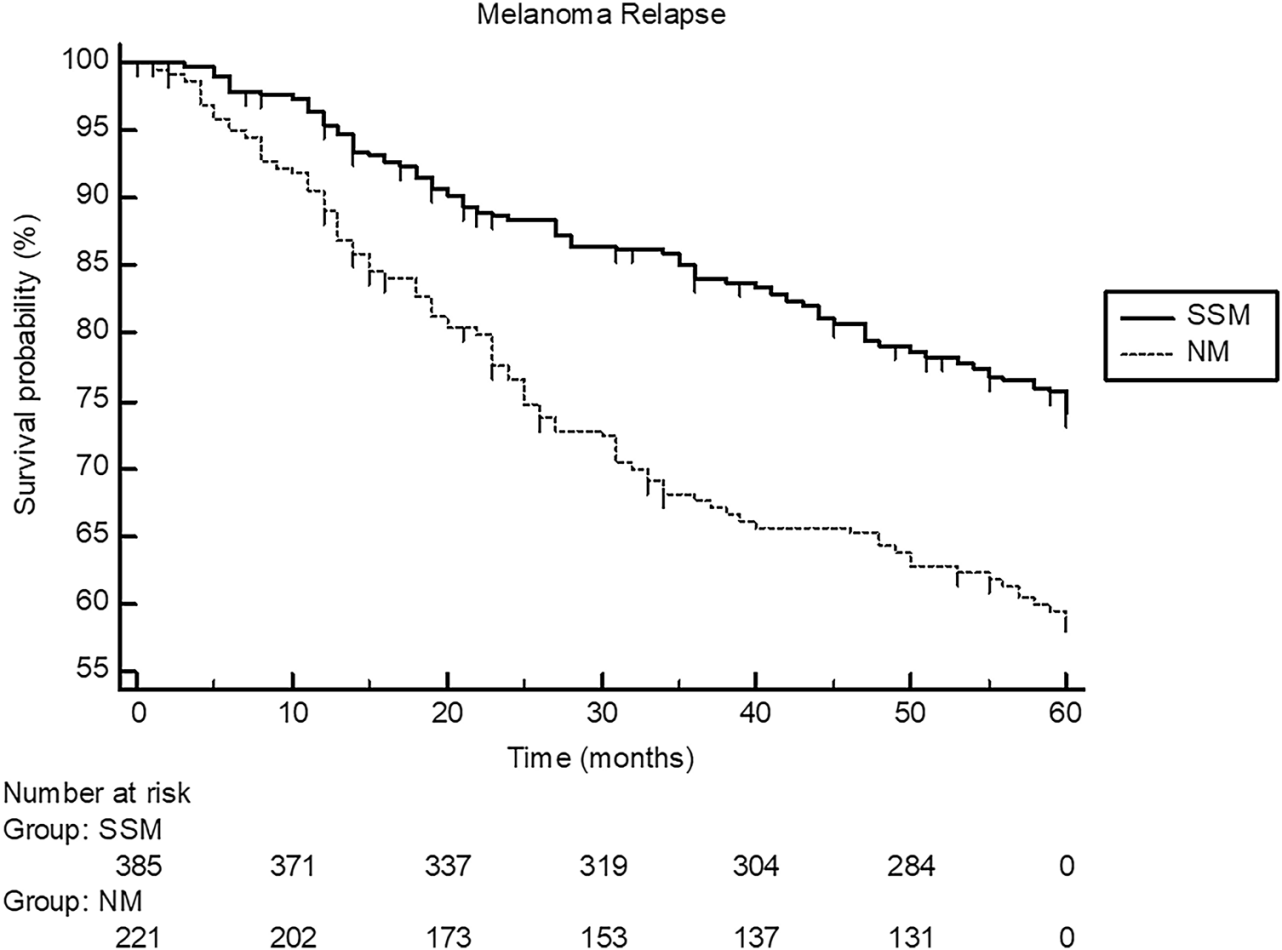
Showing the 5-year Kaplan–Meier curves for melanoma relapse in patients with nodular melanoma (NM; *n* = 228) and patients (*n* = 396) with superficial spreading melanoma (SSM). Disease relapse significantly occurred more often in patients with NM (log-rank test: *P* < 0.0001; hazard ratio: 1.92, 95% confidence interval 1.41–2.61)

**Fig. 2:**
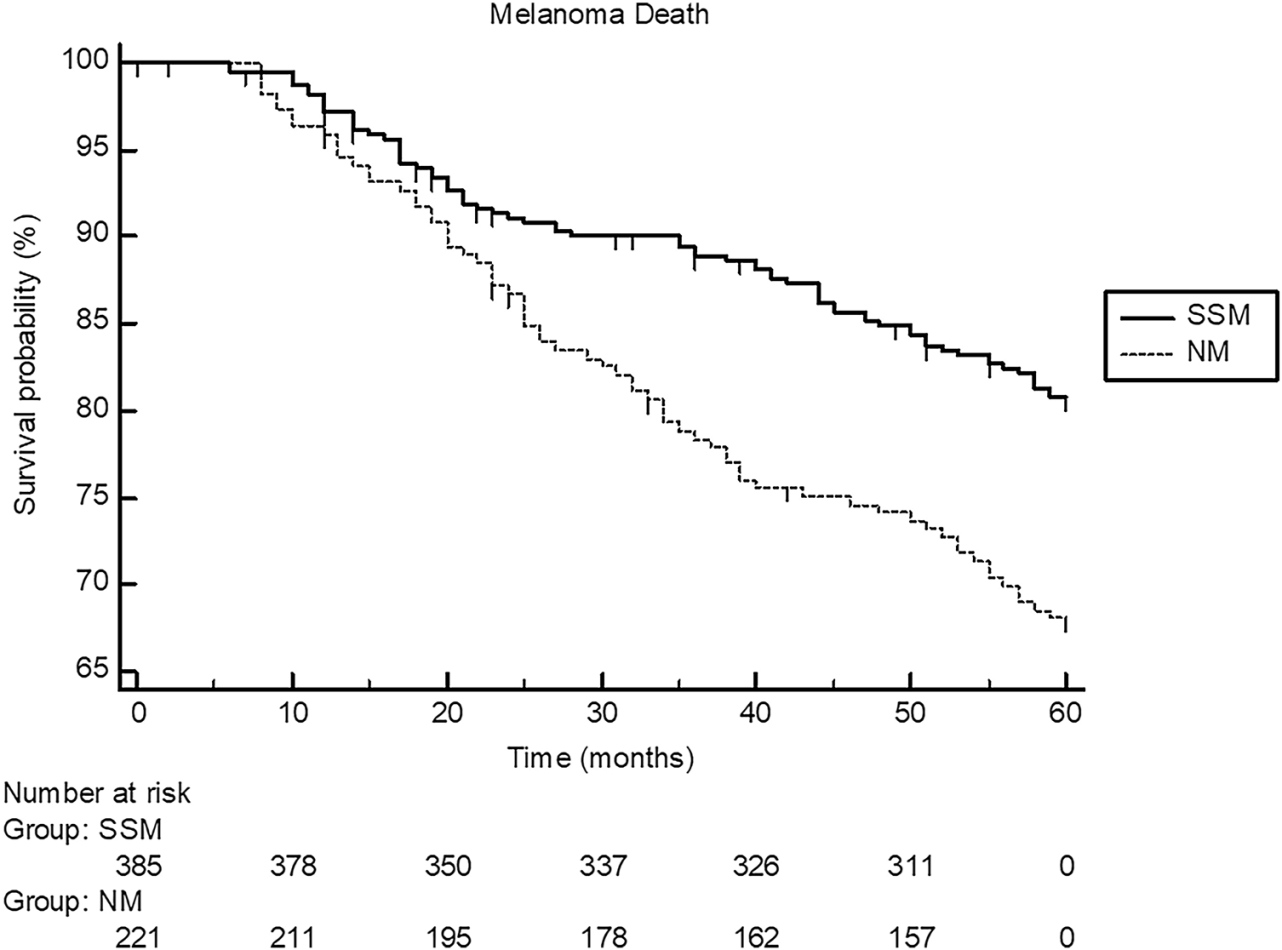
Showing the 5-year Kaplan–Meier curves for melanoma death in patients with nodular melanoma (NM; *n* = 228) and patients (*n* = 396) with superficial spreading melanoma (SSM). Melanoma-specific death significantly occurred more often in patients with NM (log-rank test: *P* < 0.0004; hazard ratio: 1.90, 95% confidence interval 1.33–2.66)

When compared to patients with SSM, patients with NM had less likely lower Clark level (*P* < 0.0001), higher tumor thickness (*P* < 0.0001), less likely tumor regression (*P* = 0.019), more often ulcerated tumors (*P* = 0.0008), and less likely a history of pre-existing lesions such as nevus (*P* < 0.0001). As also demonstrated in Table 1 more in detail, patients with NM had more frequently a positive SLNB (*P* < 0.0001), more often higher disease stage at primary diagnosis (*P* < 0.0001), and more frequently adjuvant therapy with interferon (*P* < 0.0001) as compared to patients with SSM patients. Within a 5-year follow-up, we observed significantly more disease relapses (*P* < 0.0001; hazard ratio: 1.92, 95% confidence interval 1.41–2.61; Fig. 1) and deaths (*P* = 0.0004; hazard ratio: 1.90, 95% confidence interval 1.33–2.66; Fig. 2) in NM patients than in SSM patients.

On univariate analysis, disease relapse in NM patients was significantly associated with a positive SLNB (*P* = 0.0021), with higher tumor thickness (*P* = 0.0003), and higher Clark level (*P* = 0.013). Positive SLNB status in NM patients was significantly associated with male sex (*P* = 0.0091), tumor thickness (*P* = 0.0055), and tumor location on the upper limbs (*P* = 0.022). These factors also remained in the logistic regression model as independent predictors for positive SLNB status in NM patients (tumor thickness; *P* = 0.0038, odds ratio: 2.4, 95 CI 1.3–4.3; male sex; *P* = 0.0066, odds ratio: 2.2, 95% CI 1.2–3.8; tumor location on the upper limbs, *P* = 0.037, odds ratio: 2.2, 95% CI 1.1–4.6). Using a logistic regression model for the total study population with respect to disease relapse and melanoma-specific death, we found that higher tumor thickness was the only factor remaining significant in the model with odds ratios of 3.1 (95% CI 2.1–4.4, *P* < 0.0001) and 1.2 (95% CI 1.1–1.4, *P* = 0.0011), respectively.

On univariate analysis, disease relapse in NM patients was significantly associated with higher Clark levels (*P* = 0.013), high-risk tumor thickness (*P* = 0.0003), positive SLNB (*P* = 0.0021), and absence of tumor regression (*P* = 0.0061). On multivariate analysis, disease relapse in NM patients was independently predicted by tumor thickness (*P* = 0.0077; odds ratio: 2.4, 95% CI 1.2–4.3) and positive SLNB (*P* = 0.015; odds ratio: 2.1 95% CI 1.2–3.6). Melanoma-specific death was significantly associated with higher Clark level (P = 0.017), male sex (*P* = 0.0081), and high-risk tumor thickness (*P* = 0.024). Multivariate analysis revealed that melanoma-specific death of NM patients was independently predicted by male sex (*P* = 0.013; odds ratio: 2.1, 95% CI 1.2–3.9) and tumor thickness (*P* = 0.020; odds ratio: 2.1, 95% CI 1.1–4.1). Histologic regression also remained in the logistic regression model as a significant independent negative predictor of NM death (*P* = 0.031; odds ratio: 0.21, 95% CI 0.051–0.87).

## Discussion

The unfavorable outcome of NM compared to SSM has been studied among many cohorts, comprising single-center studies and large regional, national, and international data bases (Greenwald et al. 2012; Lattanzi et al. 2019; Dessinioti et al. 2018; Chamberlain et al. 2002; Pollack et al. 2011). Nonetheless, it is still under debate as to what extent the poor prognosis of NM is just driven by well-established factors such as higher tumor thickness. The transformation of melanocytes in the epidermis starts with radial growth and gradually goes over into vertical growth corresponding to the previously proposed model of linear MM progression. Whereas it is impossible to differ the vertical growth phases of NM and SSM, it seems to be possible now to describe differences between these MM subtypes at the molecular level (Lattanzi et al. 2019, Dessinioti et al. 2018, Balch 2009). Recently, Lattanzi et al. (Lattanzi et al. 2019) performed a very large study (*n* = 118.508) using the population-based Surveillance, Epidemiology and End Results (SEER) data from 1973 to 2012. They showed that compared with SSM, NM was a statistically significant risk factor for overall mortality (Lattanzi et al. 2019). As in our study, stage IV patients at first diagnosis and other melanoma subtypes besides SSM and NM were not included in the analysis. Recently, Allais et al. (Allais et al. 2020) determined the difference in 5-year relative survival in patients with NM and SSM at the same Breslow depth and TNM stage obtained from the SEER register. They showed that 5-year relative survival was worse in NM patients as compared to patients with SSM, particularly in T1b, T2a, and T2b melanomas. Accordingly, Lindholm et al. (2004) included 6191 stage I and II patients with SSM, NM, and other subtypes between 1990 and 1999. They observed a hazard ratio for melanoma-specific death of 1.35 (95% CI = 1.08–1.70) for NM compared to SSM. In contrast, El Sharouni et al. (EL Sharouni et al. 2020) studied almost 50.000 MM patients, including approximately 80% primary SSM and about 15% primary NM. They observed that NM patients with tumors greater than 1 mm tumor thickness did not show worse survival than SSM patients with tumors greater than 1 mm. Only patients with thinner NM showed increased risk for melanoma death when compared to SSM. Although they found that thin NM was statistically significantly associated with worse survival, the hazard ratio was only 1.06 (95% CI = 1.01–1.12). A type I error may be considered given the large sample size investigated. In an international investigation published by Dessinioti et al. (Dessinioti et al. 2018), 20.132 NM and SSM patients with thin tumors (≤ 1 mm) were analyzed. In line with the data of El Sharouni et al. (Sharouni et al. 2020), they found that thin NM, particularly between 0.8 and 1 mm tumor thickness, were associated with worse prognosis when compared to thin SSM tumors (Sharouni et al. 2020; Green et al. 2012).

In contrast, Robsahm et al. (Robsahm et al. 2018) did not observe that NM subtype is a significant independent predictor for melanoma-specific survival (*n* = 5010, 2008–2012). They found an insignificant hazard ratio of 1.01 (95% CI = 0.79–1.29) for NM. Similar to most other studies discussed herein, we studied a MM population treated prior to the era of novel therapies such as immune checkpoint inhibitors and targeted therapies. Our data are in line with the results of Robsahm et al. (Robsahm et al. 2018). Using a logistic regression model with respect to melanoma-specific death, we found that high tumor thickness and male sex was the only factor remaining significant in the model. Notably, NM subtype was significant only on univariate analysis. Moreover, we confirmed that melanoma-specific death in patients with NM was significantly associated with higher Clark level, male sex, and high-risk tumor thickness. On multivariate analysis, disease relapse in NM patients was independently predicted by high-risk tumor thickness and positive SLNB (Pizzichetta et al. 2017; Faut et al. 2017; Barnhill et al. 2020).

In accordance with data of Dessinioti et al. (Dessinioti et al. 2018), we also observed that histologic regression is a significant independent negative predictor of NM death. Indeed, the absence of regression is nowadays considered a high-risk characteristic for unfavorable outcome (Ribero et al. 2015; Pan et al. 2017). Furthermore, we observed that our patients with NM more frequently reported that their melanoma was not associated with a precursor lesion such a nevus. A finding that was also reported by Dessinioti et al. (Dessinioti et al. 2018), who performed a large international study. Nevus remnants or de novo melanomas are more frequently associated with the NM subtype than SSM (Dessinioti et al. 2018; Tas and Erturk 2016). Indeed, the main limitations of the present study are the retrospective design and relatively small sample size when compared to large-scale national or even international investigations. A limitation regarding the statistics is that no corrections for multiple comparisons have been made. However, this was a limitation of almost all previous studies in this field (Dessinioti et al. 2018).

In conclusion, we did not observe that NM subtype was a significant independent predictor for disease relapse or melanoma-specific death. Higher tumor thickness was the most important prognostic factor for patients with NM. Among the well-known prognostic factors such as tumor thickness and male sex, NM is also associated with other unfavorable factors such as absence of regression.
